# Editorial: Neuromodulation in olfaction, volume II

**DOI:** 10.3389/fncel.2023.1146770

**Published:** 2023-02-02

**Authors:** Carl Edward Schoonover, Andrew Jacob Pixley Fink, Cindy Poo, Qi Yuan

**Affiliations:** ^1^Zuckerman Institute, Columbia University, New York, NY, United States; ^2^Allen Institute for Neural Dynamics, Allen Institute, Seattle, CA, United States; ^3^Champalimaud Research, Champalimaud Foundation, Lisbon, Portugal; ^4^Division of Biomedical Sciences, Faculty of Medicine, Memorial University of Newfoundland, St. John's, NL, Canada

**Keywords:** olfaction, neuromodulation, slice physiology, pharmacology, learning

The physiology of the olfactory system is shaped by a diversity of modulatory inputs. These include cholinergic projections from the basal forebrain, noradrenergic projections from the locus coeruleus, and serotonergic projections from raphe nuclei, as well as neuropeptidergic projections carrying orexin-A and oxytocin from the hypothalamus. A complete understanding of the neural basis of olfaction therefore requires knowledge of the conditions under which these modulatory regions are engaged as well as how they influence neurophysiology in the olfactory system. In turn, the relative tractability and accessibility of the olfactory system positions it as a fruitful model for elucidating the effects and mechanisms of neuromodulation.

The second volume of *Neuromodulation in Olfaction* explores how several of these aforementioned modulatory inputs influence the processing of olfactory stimuli. Regions studied include the olfactory bulb (the first stage of processing downstream of the olfactory sensory neurons in the periphery), the piriform cortex (a three-layered paleocortical region, and a major target of the olfactory bulb), and the basolateral amygdala (the division of the amygdala that receives the bulk of its sensory information, including a strong projection from the piriform). We hope this collection of original research articles and perspectives contributes to the understanding of neuromodulatory and olfactory systems in the context of cellular physiology, neural circuits, and behavior.

## Neuromodulation in slice physiology

Potts and Bekkers employ whole-cell patch clamp recordings in acute slices of mouse olfactory cortex to characterize how dopamine modulates the intrinsic excitability of piriform neurons. Dopamine has no effect on most classes of excitatory and inhibitory cell types. However, dopamine raises the intrinsic excitability of parvalbumin-expressing (inhibitory) interneurons by raising their resting potential and increasing their input resistance in a mechanism mediated by D1, but not D2, dopamine receptors.

Schubert et al. knock out the adenosine 1 receptor (A_1_R) in neurons to study the modulation of mitral and granule cells in the olfactory bulb by adenine nucleotides. They find that adenosine modulates dendro-dendritic signaling in these cells *via* A_1_R, and hyperpolarizes mitral cells, potentially improving their signal-to-noise ratio. However, this effect is not sufficient to overcome excitotoxicity in a model of inflammation-induced hyper-excitability, and consequent impairment of olfactory acuity.

Awasthi et al., employ whole-cell patch clamp recordings in acute slices of rat piriform cortex to study how prior rule learning *in vivo* affects the excitability of cortical pyramidal neurons. Rats trained to perform a difficult odor discrimination task subsequently perform better on pairs of novel odors. This rule learning is accompanied at the biophysical level by a decrease in the amplitude of the action potential after hyperpolarization, which the authors show results from reduced conductance of the M-current mediated by muscarinic cholinergic receptors.

Hu et al. employ whole-cell patch clamp recordings in acute slices of mouse olfactory bulb to characterize how noradrenaline modulates hyperpolarization-activated currents (I_h_) in granule cells. They find that noradrenaline acts on α_2_-adrenergic receptors, reducing I_h_ and thereby increasing dendritic excitability. Thus the locus coeruleus can tune, in a state-dependent manner, the lateral inhibition effected by granule cells onto neighboring mitral cells.

## Neuromodulation in behavior

Omoluabi et al., employ optogenetic control of the locus coeruleus (LC), combined with pharmacological block of adrenoceptors in the basolateral amygdala (BLA) in rats to study how LC conveys valence information to the BLA. They find that phasic activation of the LC conveys a positive-valence signal *via* both α_1_- and β-adrenoceptors. In contrast, they find that tonic activation of the LC conveys negative-valence signal *via* β-adrenoceptors alone. This mechanism is validated in reward- and punishment-based odor learning experiments.

Zhou et al. employ fiber photometry in mice to compare the habituation profiles of cholinergic and GABAergic neurons in the horizontal limb of the diagonal band of Broca. Both of these subpopulations make long range projections to olfactory centers and have been implicated in habituation of both behavior and stimulus-induced neuronal responses in olfactory bulb and in cortex. The authors find that both cholinergic and GABAergic neurons habituate when odorant stimuli are presented passively. However when the animals are engaged in a go/no-go task only GABAergic neurons habituate, whereas cholinergic activity is modified by learning in a valence-specific manner.

## Perspectives

In a Mini-Review, Rajani and Yuan consider the common but still unexplained finding that Alzheimer's disease (AD) patients experience olfactory deficits in the pre-clinical stage of the disease. In a recent rat model of AD, pretangle tau—a soluble precursor of neurofibrillary tangles—is expressed initially in the locus coeruleus (LC) and leads to degeneration of LC fibers in the piriform. These rats exhibit a loss in olfactory acuity leading the authors to propose that LC degeneration during early stages of AD may account for olfactory deficits in patients.

In a Perspective, Stensola and Stensola survey how the olfactory cortex processes odorant stimuli, and how novel experience modifies its activity by engaging mechanisms mediated in part by neuromodulatory systems. They then argue that this cortex's recurrent circuitry and proximity to the sensory periphery render it an attractive model for studying categorical learning—the faculty, acquired through experience, to efficiently group distinct sensory stimuli.

## Author contributions

All authors listed have made a substantial, direct, and intellectual contribution to the work and approved it for publication.

